# Effects of a Glutamine and Arginine Combination on the Inflammatory Response in a Lipopolysaccharide‐Induced Sepsis Model at Different Time Points of Supplementation

**DOI:** 10.1155/ccrp/9960244

**Published:** 2026-06-12

**Authors:** Maulydia Maulydia, Nancy Margarita Rehatta, Subijanto Marto Soedarmo, Widjiati Widjiati

**Affiliations:** ^1^ Department of Anesthesiology and Reanimation, Faculty of Medicine, Universitas, Airlangga, Jl. Mayjen Prof. Dr. Moestopo 47, Surabaya, 60131, Indonesia; ^2^ Faculty of Medicine, Petra Christian University, Jl. Siwalankerto 121-131, Surabaya, 60236, Indonesia, petra.ac.id; ^3^ Department of Child, Faculty of Medicine, Universitas Airlangga, Jl. Mayjen Prof. Dr., Moestopo 47, Surabaya, 60131, Indonesia, unair.ac.id; ^4^ Department of Veterinary Science, Faculty of Veterinary Medicine, Universitas Airlangga, Jl. Dharmahusada Permai 1, Surabaya, 60115, Indonesia, unair.ac.id

**Keywords:** arginine, glutamine, IL-8, MMP-8, sepsis

## Abstract

Activation of the NF‐κB signaling pathway in sepsis plays a crucial role in the expression of TNF‐α, IL‐8, and MMP‐8, which can further exacerbate the condition. The management of sepsis requires complex strategies. Glutamine and arginine are amino acids that can modulate inflammatory processes and enhance immune function, and their levels are often deficient in sepsis. This study aimed to analyze the effects of glutamine and arginine combination (GAC) on TNF‐α, NF‐κB, IL‐8, and MMP‐8 expressions in sepsis. Seventy‐two *Rattus norvegicus* were divided into three main intervention groups, each of which was further divided into three subgroups that received therapy initiated at different time points (6 h, 24 h, and 48 h) after the intervention. Therapy was administered once daily for three consecutive days. Termination was performed 2 h after the final therapy, after which jejunal tissues were collected. The administration of GAC resulted in different cells expressing IL‐8 (*p* = 0.026) in the 24‐h treatment group and MMP‐8 (*p* < 0.001) in the 6‐h treatment group. No differences were found in cells expressing TNF‐α, NF‐κB, and IL‐8 in the 6‐h treatment group (*p* > 0.05); TNF‐α, NF‐κB, and MMP‐8 in the 24‐h treatment group (*p* > 0.05); and TNF‐α, NF‐κB, IL‐8, and MMP‐8 in the 48‐h treatment group (*p* > 0.05). The supplementation of GAC demonstrated different cells expressing IL‐8 (in therapy started at 24 h) and MMP‐8 (in therapy started at 6 h). Future studies should be conducted on dose evaluation over a longer research duration to validate more specific results.

## 1. Introduction

Multiple causes of disease (MoCD) had a mortality rate of 46.7 per 1,000,000 among the population aged 0–85 years due to abdominal sepsis between 2004 and 2018 [1]. Sepsis is characterized by life‐threatening organ dysfunction and arises as a result of infection or systemic inflammatory response (SIRS) [2, 3]. Sepsis modeling using cecal ligation and puncture (CLP) demonstrated strong activation of the NF‐κB signaling pathway after 6 h of exposure [4]. NF‐κB plays an important role in inflammatory responses through M1 macrophage activation and the production of proinflammatory cytokines such as TNF‐α [5]. TNF‐α is a proinflammatory cytokine mainly produced by macrophages that contributes to immune and inflammatory responses through its interaction with other cytokines such as IL‐6 and IL‐8 [6]. The inhibition of NF‐κB activation has been shown to reduce TNF‐α levels and prevent MMP‐8 expression, resulting in improved survivability under septic conditions [7, 8]. The peak of NF‐κB activity was observed at a 24‐h timestamp [9].

Early identification and appropriate management are crucial for improving outcomes in sepsis. Fluid resuscitation, vasopressor administration, antimicrobial therapy, pathogen identification, nutritional support, and other supportive therapies are essential components of sepsis treatment [10]. Nutrients play various roles in the management of sepsis patients, including improving pre‐epithelial functions, preventing villous atrophy of the intestinal mucosa, maintaining the intestinal epithelium, stimulating secretion of brush border enzymes, enhancing immune functions, and maintaining the tight junctions of epithelial cells [11]. The European Society for Clinical Nutrition and Metabolism (ESPEN) recommends that in patients with a critical condition where oral nutritional intake is almost impossible, early enteral nutrition should be started within the first 48 h and prioritized over parenteral nutrition [11]. Among the nutrients involved in sepsis management, amino acids such as glutamine and arginine have gained particular attention for their roles in modulating immune and inflammatory responses [12].

Glutamine can be rapidly depleted from muscles in catabolic conditions such as sepsis; thus, it is categorized as a conditionally essential nutrient. In sepsis, cytokines and glucocorticoids mediate increased hepatic glutamine uptake, while glutamine utilization by the intestines is reduced, a response that may correlate with reduced insulin‐like growth factor‐I (IGF‐I), a characteristic of sepsis [13]. Glutamine supplementation has emerged as a potential strategy for mitigating intestinal inflammation by inhibiting NF‐κB and STAT and suppressing the expression of inflammatory cytokines, including IL‐6, TNF‐α, and IL‐8 [14].

Arginine deficiency in sepsis is mainly due to decreased arginine levels associated with specific changes in arginine metabolism that are directly linked to intense catabolism. An increasing amount of evidence suggests that during the evolution of the immune system, arginine has been selected as the key regulator of the immune system. An adequate arginine supply has long been associated with increased immune responses. In addition to serving as a building block for protein synthesis, arginine also functions as a substrate for different metabolic pathways that significantly influence the biology of immune cells, particularly the immunobiology of macrophages, dendritic cells, and T cells. Arginine availability, synthesis, and catabolism are interconnected aspects of immune responses, and these adjustments can determine different proinflammatory or anti‐inflammatory immune outcomes [15]. Arginine supplementation can reduce TNF‐α and IL‐8 levels [16, 17].

The administration of glutamine and arginine remains controversial. Glutamine administration is beneficial in reducing inflammation, protecting organs from sepsis‐induced damage, addressing certain deficiencies to reduce mortality and bacterial translocation, and increasing mucosal thickness and antioxidant levels. On the other hand, arginine administration has beneficial effects in reducing bacteremia and the mortality rate of sepsis patients and in preventing T‐lymphocyte dysfunction. Arginine is safe for use via enteral and parenteral routes. However, the effectiveness of glutamine and arginine and their safety at higher doses still need to be confirmed concerning their potential adverse effects. In particular, high doses of arginine may cause adverse effects in patients with critical conditions and septic shock [18].

Until now, studies investigating the effects of the glutamine and arginine combination (GAC) in sepsis have been limited, particularly those evaluating its influence on TNF‐α, NF‐κB, IL‐8, and MMP‐8 expressions at different time points of administration. Hence, this study aimed to assess these effects following GAC supplementation, which has not been previously reported.

## 2. Materials and Methods

### 2.1. Animal and Experimental Design

This study used healthy and active male *Rattus norvegicus* Wistar strain rats aged 12–14 weeks, with body weights ranging from 150 to 200 g, which had never been used in previous experiments. Male rats were used to reduce hormonal variability; however, this limits the generalizability of the findings, as sex differences are known to influence sepsis outcomes. The rats were obtained from a certified institutional animal facility and were confirmed to be healthy and free from infectious diseases. Rats showing signs of poor health (droopy eyes, flattened nose or cheeks, inward curved ears, and tense whiskers), aggressive behavior, loss of appetite, or death before the intervention were excluded. Rats that died during the intervention or before anesthesia were considered dropouts and excluded from the study. The sample size was calculated using Lemeshow’s formula for analytical studies, assuming a significance level (α) of 0.05, a statistical power of 80%, and an expected moderate effect size based on previous studies investigating inflammatory markers in sepsis models. This resulted in a minimum of eight animals per group. There were nine treatment groups, consisting of three main intervention groups, each further divided into three subgroups based on treatment time points.

The main groups were designated as follows: Group A (rats receiving lipopolysaccharide (LPS) injection and dextrose therapy), Group B (rats receiving LPS injection and GAC therapy), and Group C (rats receiving NaCl and dextrose therapy). Groups A and C served as control groups, with Group A as the positive control (LPS control) and Group C as the negative control (NaCl control). These two control groups were compared with Group B, the intervention group, to evaluate the effects of GAC administration on sepsis.

Each main group was further divided according to treatment time points of 6 h, 24 h, and 48 h after initiation of treatment. In total, seventy‐two rats were included in this study.

### 2.2. Animal Care and Acclimatization

Rats that met the criteria were randomly selected and placed in separate cages. Once 72 rats had been obtained, random allocation into groups was carried out by marking the rats with colored paint on specific body parts to indicate group numbering.

Experimental animals were kept at a room temperature inside plastic cages capped with wire mesh and lined with rice husks as bedding. Each cage contained eight rats, equipped with sawdust as feed and clean water. The cages were placed inside an isolated, locked room with appropriate air ventilation and lighting. The experimental animals were acclimatized for a week before the experiment. Experimental animal conditions and body temperature were measured during the experiment using the murine sepsis score (MSS).

### 2.3. Experimental Procedure

**FIGURE 1:**
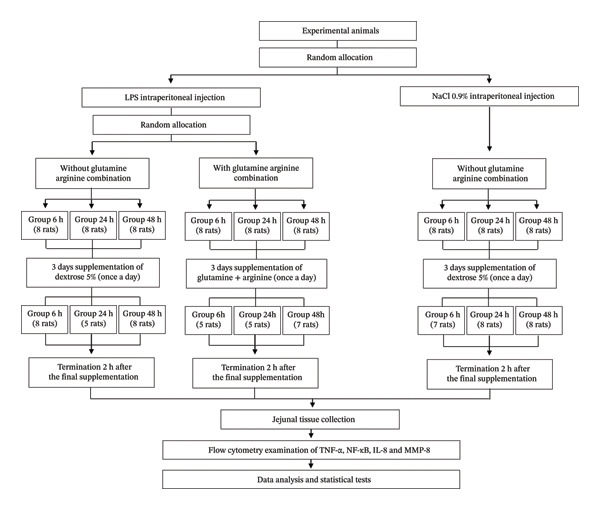
Research flowchart.

Sepsis modeling was performed in the positive control group (Group A) and the intervention group (Group B) using LPS 2 mL/kg BW injection, while the negative control group (Group C) received 2 mL/kg BW NaCl 0.9% injection. Injections were performed intraperitoneally on the lower side of the abdomen. Each group (i.e., Groups A, B, and C) was divided into three groups based on the starting time of therapy, namely, 6, 24, and 48 h after LPS or NaCl injections. The selected time points (6 h, 24 h, and 48 h) were based on the known temporal activation of the NF‐κB signaling pathway and the clinical window for early nutritional intervention. The rationale for the GAC supplementation time points was based on previous studies demonstrating that the inhibition of NF‐κB activation can reduce TNF‐α levels, which in turn may decrease IL‐8 activation and prevent MMP‐8 expression. Additionally, this timing was selected considering the strong activation of the NF‐κB signaling pathway observed as early as 6 h after sepsis induction, with peak activity at 24 h, as well as the ESPEN recommendation to administer early enteral nutrition within the first 48 h. Dextrose therapy administered to Groups A and C consisted of 1 mL of dextrose 5%. Group B received GAC therapy consisting 1:1 of L‐glutamine and L‐arginine. The dosages adopted from Bakir et al. consisted of 250 mg/kg/d of L‐glutamine and 250 mg/kg/d of L‐arginine [13]. GAC was prepared by dissolving 50 mg glutamine and 50 mg arginine in 1 mL of distilled water for rats weighing 200 g, which were administered with a 1‐mL syringe. Administration of the solution to experimental animals weighing > 200 g was adjusted based on the weight of each rat. Every experimental animal received therapy once a day for three consecutive days at the same time as that on the first day. A schematic of the research flowchart is presented in Figure 1.

### 2.4. Sample Collection and Analysis

The termination process was performed 2 h after the final therapy on the third day. Experimental animals were euthanized by ether inhalation using a closed chamber system (5 mL ether) with a short exposure time (2‐3 min), in accordance with the institutional laboratory protocol at the time of the experiment. Although current guidelines recommend alternative agents due to safety concerns, all procedures were performed under ethical approval. Rats that showed no movement were confirmed dead and immediately dissected to collect jejunal tissue. The collected jejunal tissue was mechanically dissociated and filtered to obtain a single‐cell suspension prior to intracellular staining and flow cytometry analysis. The resulting cell suspension was then subjected to intracellular staining and analysis by flow cytometry to assess the expression of TNF‐α, NF‐κB, IL‐8, and MMP‐8. Flow cytometry data were analyzed using IBM SPSS Statistics 21 with multivariate analysis of variance (MANOVA) to evaluate the effect of glutamine–arginine combination administration on the percentage of cells expressing TNF‐α, NF‐κB, IL‐8, and MMP‐8. Multivariate significance was evaluated using Pillai’s Trace test. Prior to conducting MANOVA, the assumptions of multivariate normality and homogeneity of variance–covariance matrices were assessed. Although some variability was observed, MANOVA was considered robust given the balanced design and comparable group sizes. Multivariate test statistics (Pillai’s Trace and corresponding F‐values) were considered in the analysis; however, only *p* values are presented for clarity.

This study was approved by the Ethical Committee under the ethical clearance number 2.KE.027.03.2021. Additionally, this study was designed and reported in accordance with the 10 essential items of the ARRIVE Guidelines 2.0 for animal studies. The completed checklist is provided in the supporting informations (Supporting File 1).

## 3. Results and Discussion

All data are presented as the percentage of marker‐positive cells among total viable lymphocytes. Not all rats in the experimentation groups survived. The number of surviving rats in Group A (LPS + dextrose) was eight for the 6‐h administration, five for the 24‐h administration, and eight for the 48‐h administration. In Group B (LPS + GAC), the number of surviving rats was five for the 6‐h administration, five for the 24‐h administration, and seven for the 48‐h administration. Meanwhile, in Group C (NaCl + dextrose), the number of surviving rats was 7 for the 6‐h administration and 8 after the 24‐h and 48‐h administrations, respectively.

Sepsis is known to trigger ischemia‐reperfusion (I/R) injury in the intestine and increase intestinal or systemic MMP‐8 expression. Peak MMP‐8 expression occurs 3 h after ischemia [19]. Gastrointestinal mucosal damage, such as in the colon, has been found to exhibit increased collagenase activity, which correlates with inflammation intensity [20]. MMP‐8 is assumed to be the primary collagenase involved in the mucosal damage process [21]. Increased MMP‐8 as the initial response to injuries was also found in a blunt chest injury model, occurring 1–6 h after the trauma [22]. In this study, we found a significant difference in the percentage of cells expressing MMP‐8 in the treatment group that received a GAC 6 h after LPS injection. The administration of the GAC started 6 h after the LPS injection showed a lower percentage of MMP‐8‐expressing cells compared with other groups that did not receive the GAC as shown in Table 1.

**TABLE 1:** Percentage (%) of marker‐positive cells at 6‐h administration.

Variables	Group A (LPS + dextrose)	Group B (LPS + GAC)	Group C (NaCl + dextrose)	*p* ^a^	*p* ^b^
TNF‐α	4.45 ± 1.94	3.74 ± 1.55	6.44 ± 4.72	0.502	0.250
NF‐κB	1.61 ± 1.69	0.98 ± 1.21	7.70 ± 9.24	0.487	0.142
IL‐8	17.87 ± 6.91	23.62 ± 14.35	14.07 ± 7.88	0.347	0.167
MMP‐8	13.86 ± 15.03	8.31 ± 13.37	34.31 ± 18.98	0.515	0.026^∗^

*Note:* Data are presented as mean ± standard deviation (SD) of the percentage of marker‐positive cells among total viable lymphocytes; *p* values represent pairwise comparisons between groups following MANOVA.

^a^ = comparison between Group A (LPS + dextrose) and Group B (LPS + GAC).

^b^ = comparison between Group B (LPS + GAC) and Group C (NaCl + dextrose).

^∗^ *p* < 0.05 indicates statistical significance.

A significant difference was found in the percentage of cells expressing IL‐8 between GAC and NaCl control group at the 24‐h administration time (Table 2). IL‐8 is a proinflammatory mediator expressed in many cells, including monocytes, macrophages, dermal fibroblasts, endothelial cells, keratinocytes, mesangial cells, and several human tumor cells. IL‐8 is secreted by the infected intestinal epithelial cells [23]. It functions to activate and guide neutrophils to the inflamed area; thus, IL‐8 is known as a *neutrophil-activating chemotactic cytokine*. Glutamine supplementation can suppress the increase in proinflammatory IL‐8 expression after LPS administration [24]. Similarly, arginine supplementation at a dose of 8 mM was shown to be effective in reducing IL‐8 expression 6 h after LPS exposure [17]. This study has different findings, where the administration of GAC 24 h after LPS injection resulted in a significant increase in the percentage of cells expressing IL‐8, with a tendency that the group receiving GAC had a higher mean percentage of cells expressing IL‐8 than the control groups. The higher IL‐8 expression observed in the GAC treatment group may reflect the dual role of IL‐8 as a proinflammatory mediator while also contributing to the resolution of inflammation and tissue healing [25]. IL‐8’s anti‐inflammatory activities are based on its role in preventing leukocyte adhesion to active endothelial cells. IL‐8 plays a crucial role in inflammation and wound healing. IL‐8 can recruit T cells and nonspecific inflammatory cells to the inflamed area by activating neutrophils [26].

**TABLE 2:** Percentage (%) of marker‐positive cells at 24‐h administration.

Variables	Group A (LPS + dextrose)	Group B (LPS + GAC)	Group C (NaCl + dextrose)	*p* ^a^	*p* ^b^
TNF‐α	4.91 ± 3.11	1.61 ± 1.00	2.31 ± 1.59	0.054	0.401
NF‐κB	1.37 ± 0.87	2.08 ± 3.76	0.73 ± 0.71	0.689	0.331
IL‐8	14.49 ± 5.72	20.42 ± 3.59	6.97 ± 2.81	0.085	< 0.001^∗^
MMP‐8	8.94 ± 8.29	9.23 ± 15.27	8.12 ± 8.94	0.971	0.871

*Note:* Data are presented as mean ± standard deviation (SD) of the percentage of marker‐positive cells among total viable lymphocytes; *p* values represent pairwise comparisons between groups following MANOVA.

^a^ = comparison between Group A (LPS + dextrose) and Group B (LPS + GAC).

^b^ = comparison between Group B (LPS + GAC) and Group C (NaCl + dextrose).

^∗^ *p* < 0.05 indicates statistical significance.

**FIGURE 2:**
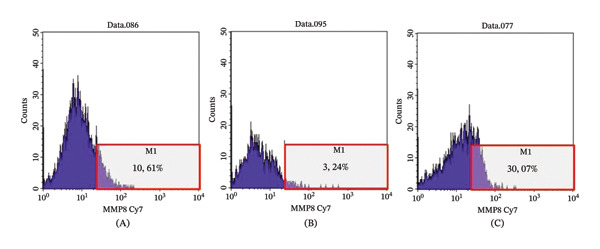
Results of flow cytometry analysis of the percentage of cells expressing MMP‐8 at the 6‐h administration time. (A) LPS control group (LPS + dextrose); (B) glutamine–arginine (LPS + GAC); and (C) NaCl control group (NaCl + dextrose).

**FIGURE 3:**
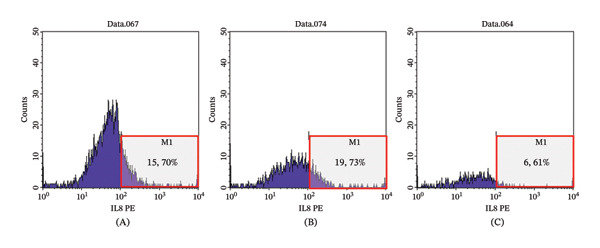
Results of flow cytometry on the percentage of cells expressing IL‐8 at the 24‐h administration time. (A) LPS control group (LPS + dextrose), (B) glutamine–arginine (LPS + GAC), and (C) NaCl control group (NaCl + dextrose).

The results of the flow cytometry analysis supporting Tables 1 and 2 are presented in Figures 2 and 3. These figures represent the percentage of living cells in the lymphocytes. Figure 2 illustrates the results of the flow cytometry analysis after 6‐h treatment. The figure indicates that the lowest percentage of cells expressing MMP‐8 was observed in the GAC group. Of the 100% living cells found in lymphocytes, 3.24% expressed MMP‐8 in the GAC group, 10.61% expressed MMP‐8 in the LPS control group, and 30.07% expressed MMP‐8 in the NaCl control group. Meanwhile, the results of the flow cytometry analysis at the 24‐h administration time showed that the highest percentage of IL‐8 expressing cells was found in the GAC group. Figure 3 shows that of the 100% living cells found in lymphocytes, 6.61% cells expressing IL‐8 were found in the NaCl control group, 15.70% cells expressing IL‐8 were found in the LPS group, and 19.73% cells expressing IL‐8 were found in the GAC group.

In contrast to MMP‐8 and IL‐8, as illustrated in Tables 1, 2, and 3, the number of cells expressing TNF‐α and NF‐κB was not significantly different between the GAC group and the control group at all time points. This finding contradicts those of previous studies that showed the effects of the GAC on TNF‐α and NF‐κB. A preliminary study on the role of GAC in trauma modeling revealed a significant decrease in TNF‐α levels [12]. Enteral supplementation of GAC administered 10 days before LPS exposure resulted in a lower serum TNF‐α level compared with supplementation with glutamine and arginine as a single substance [13]. Supplementation with a GAC administered 15 days before the experimental animals were exposed to LPS has been proven to reduce TNF‐α levels in the jejunum and ileum [13, 27]. The high standard deviations observed in some groups suggest biological variability inherent to the sepsis model. Data distribution was assessed, and no transformation was applied, as MANOVA is relatively robust to moderate deviations from normality.

**TABLE 3:** Percentage (%) of marker‐positive cells at 48‐h administration.

Variables	Group A (LPS + dextrose)	Group B (LPS + GAC)	Group C (NaCl + dextrose)	*p* ^a^	*p* ^b^
TNF‐α	5.59 ± 2.95	8.24 ± 6.26	6.02 ± 3.71	0.302	0.411
NF‐κB	18.44 ± 18.23	6.37 ± 8.22	9.58 ± 6.77	0.132	0.421
IL‐8	12.59 ± 11.83	18.70 ± 12.95	10.06 ± 10.84	0.357	0.183
MMP‐8	11.88 ± 8.48	5.28 ± 8.65	10.78 ± 11.02	0.160	0.307

*Note:* Data are presented as mean ± standard deviation (SD) of the percentage of marker‐positive cells among total viable lymphocytes; *p* values represent pairwise comparisons between groups following MANOVA. *p* < 0.05 indicates statistical significance.

^a^ = comparison between Group A (LPS + dextrose) and Group B (LPS + GAC).

^b^ = comparison between Group B (LPS + GAC) and Group C (NaCl + dextrose).

Glutamine plays a crucial role in modulating inflammatory responses by inhibiting NF‐κB activation, a key regulator of inflammation. Additionally, glutamine reduces protein phosphorylation associated with transcription factors involved in immune cell proliferation, development, and modulation [13]. A significantly low NF‐κB level was found in sepsis rats receiving glutamine supplementation [28]. In this study, the administration of a GAC did not affect cells expressing NF‐κB. This insignificant result in the observation of cells expressing NF‐κB was likely to be caused by inappropriate dose administration, resulting in an unoptimized effect on NF‐κB. Supplementation with 500‐µM arginine significantly reduces the increased expression of NF‐κB p65 after LPS exposure [29]. Supplementation with 10‐µM glutamine following LPS exposure suppressed the increased activation of the NF‐κB signaling pathway [30].

The discrepancy in TNF‐α and NF‐κB findings compared with previous studies may be attributed to several factors, including methodological differences such as the tissue type analyzed, timing of measurement, and experimental design [13, 27]. In addition, the shorter duration of supplementation in the present study may have contributed to the lack of significant effects, as previous studies have reported that GAC significantly reduced TNF‐α levels after 10–15 days of administration [13, 27]. Taken together, these findings suggest that the effects of GAC supplementation may vary depending on the duration of treatment, timing of administration, and the specific inflammatory mediators involved.

This study also utilized male rats, consistent with many preclinical sepsis studies, where male animals have traditionally been used to minimize biological variability associated with hormonal influences. However, it is well established that sex differences significantly influence sepsis progression and immune responses, with female animals often demonstrating improved outcomes due to the modulatory effects of estrogen. Therefore, the findings of this study should be interpreted within this context and may not be directly generalizable to females.

Mortality occurred in several experimental groups during the intervention period due to the severity of the sepsis model. Although the number of surviving animals differed between groups, this mortality pattern is consistent with the expected outcomes in LPS‐induced sepsis models. Unequal survival across groups may introduce selection bias and should be considered when interpreting the results. The high variability observed in several markers also reflects the biological complexity of the sepsis model. This study used a single‐sex model, which may limit generalizability, as sex differences are known to influence sepsis outcomes.

Although this study has a limitation due to the shortened supplementation duration, which could affect our findings, it could still serve as an additional reference by providing different perspective on sepsis treatment, which has not been addressed in previous studies. We focused on the experimental concept of using GAC as a therapeutic agent for sepsis, considering that, in clinical practice, patients are often already septic upon admission. This differs from previous studies, which adopted the paradigm of using GAC as a protective or preventive agent against sepsis progression by administering GAC prior to LPS exposure [13, 27]. Moreover, this study examined the effects of GAC supplementation at three different time points, which have not been reported in previous studies. As demonstrated in our findings, GAC showed beneficial effects at 6 and 24 h after LPS exposure.

## 4. Conclusions

The administration of GAC 6 h after exposure indicated a difference in the percentage of cells expressing MMP‐8, while the administration 24 h after exposure revealed differences in IL‐8 expression. There were no differences in the biomarker expression after 48 h of exposure. Further studies applying various doses over a longer duration are needed to obtain more specific findings.

## Author Contributions

Maulydia Maulydia: conceptualization, methodology, investigation, data curation, formal analysis, visualization, writing–original draft, and writing–review and editing. Nancy Margarita Rehatta: conceptualization, methodology, formal analysis, writing–original draft, and supervision. Subijanto Marto Soedarmo: methodology, formal analysis, writing–review and editing, and supervision. Widjiati Widjiati: methodology, writing–review and editing, supervision, and validation.

## Funding

This research did not receive any specific grant from funding agencies in the public, commercial, or not‐for‐profit sectors.

## Disclosure

All authors have read and approved the final manuscript and agreed to be accountable for all aspects of the work.

## Conflicts of Interest

The authors declare no conflicts of interest.

## Supporting Information

Additional supporting information can be found online in the Supporting Information section.

## Supporting information


**Supporting Information** Supporting Materials. The following supporting material is available: Supporting File 1. ARRIVE Guidelines 2.0 checklist (completed by the authors).

## Data Availability

The data used and/or analyzed during the current study are available from the corresponding author upon reasonable request.
