# Supplementary material for: Effects of a Glutamine and Arginine Combination on the Inflammatory Response in a Lipopolysaccharide‐Induced Sepsis Model at Different Time Points of Supplementation
Source: Crit Care Res Pract. 2026 Jun 12;2026:9960244. doi: 10.1155/ccrp/9960244 (PMC13261681; doi:10.1155/ccrp/9960244)
Supplement: Supplementary file 1 — Supporting Information Supporting Materials. The following supporting material is available: Supporting File 1. ARRIVE Guidelines 2.0 checklist (completed by the authors). [file CCRP-2026-9960244-s001.docx]

**ARRIVE GUIDELINES 2.0 CHECKLIST**

| **ARRIVE 2.0** | **Line number** | **Sentences** |
| --- | --- | --- |
| 1. **Study design** |  |  |
| 1. The groups being compared, including control groups. If no control group has been used, the rationale should be stated. | 83-88 | - The main groups were designated as follows: Group A (rats receiving lipopolysaccharide (LPS) injection and dextrose therapy), Group B (rats receiving LPS injection and GAC therapy), and Group C (rats receiving NaCl and dextrose therapy). Groups A and C served as control groups, with Group A as the positive control (LPS control) and Group C as the negative control (NaCl control). These two control groups were compared with Group B, the intervention group, to evaluate the effects of GAC administration on sepsis. |
| 1. The experimental unit (e.g., a single animal, litter, or cage of animals). | 94-97 | - Each rat was considered as the experimental unit. - Experimental animals were kept at a room temperature inside plastic cages capped with wire mesh and lined with rice husks as bedding. - Each cage contained eight rats, equipped with sawdust as feed and clean water. The cages were placed inside an isolated, locked room with appropriate air ventilation and lighting. |
| 1. **Sample size** |  |  |
| 1. Specify the exact number of experimental units allocated to each group, and the total number in each experiment. Also indicate the total number of animals used. | 73-74; 81-82;  89-90 | - This study used healthy and active male Rattus norvegicus Wistar strain rats aged 12–14 weeks, with body weights ranging from 150 to 200 g - There were nine treatment groups, consisting of three main intervention groups, each further divided into three subgroups based on treatment time points. - Each main group was further divided according to treatment time points of 6 h, 24 h, and 48 h after initiation of treatment. In total, seventy-two rats were included in this study. - Each group consisted of eight rats, resulting in a total of seventy-two rats used in this study. |
| 1. Explain how the sample size was decided. Provide details of any a priori sample size calculation, if done | 79-82 | - The sample size was determined a priori using Lemeshow’s formula for analytical studies, based on an expected effect size and 95% confidence level, resulting in eight rats per group. There were nine treatment groups, consisting of three main intervention groups, each further divided into three subgroups based on treatment time points. |
| 1. **Inclusion and exclusion criteria** |  |  |
| 1. Describe any criteria used for including or excluding animals (or experimental units) during the experiment, and data points during the analysis. Specify if these criteria were established a priori. If no criteria were set, state this explicitly. | 73-75; 76-78 | - All inclusion and exclusion criteria were established a priori. - This study used healthy and active male Rattus norvegicus Wistar strain rats aged 12–14 weeks, with body weights ranging from 150 to 200 g, which had never been used in previous experiments. - Rats showing signs of poor health (droopy eyes, flattened nose or cheeks, inward curved ears and tense whiskers), aggressive behaviour, loss of appetite, or death before the intervention were excluded. - In the 6-hour supplementation group, only five rats survived in group B. One rat died after LPS injection and before receiving the first glutamine–arginine gavage, while two other rats died before the second glutamine–arginine administration. In group C, seven out of eight rats survived. One rat died after NaCl injection and before receiving the first dextrose gavage. Data from these deceased animals were therefore excluded from the analysis. |
| 1. For each experimental group, report any animals, experimental units, or data points not included in the analysis and explain why. If there were no exclusions, state so. | 78-79 | - Rats that died during the intervention or before anesthesia were considered dropouts and excluded from the study. |
| 1. For each analysis, report the exact value of *n* in each experimental group. | 126-131 | - Not all rats in the experimentation groups survived. The number of surviving rats in Group A (LPS + dextrose) was eight for the 6 h administration, five for the 24 h administration, and eight for the 48 h administration. In Group B (LPS + GAC), the number of surviving rats was five for the 6 h administration, five for the 24 h administration, and seven for the 48 h administration. Meanwhile, in Group C (NaCl + dextrose), the number of surviving rats was 7 for the 6 h administration and 8 after the 24 h and 48 h administrations, respectively. |
| 1. **Randomisation** |  |  |
| 1. State whether randomisation was used to allocate experimental units to control and treatment groups. If done, provide the method used to generate the randomisation sequence. | 91-93 | - Rats that met the criteria were randomly selected and placed in separate cages. Once 72 rats had been obtained, random allocation into groups was carried out by marking the rats with colored paint on specific body parts to indicate group numbering. |
| 1. Describe the strategy used to minimise potential confounders such as the order of treatments and measurements, or animal/cage location. If confounders were not controlled, state this explicitly. | 94-98; 110-112 | - Experimental animals were kept at a room temperature inside plastic cages capped with wire mesh and lined with rice husks as bedding. Each cage contained eight rats, equipped with sawdust as feed and clean water. The cages were placed inside an isolated, locked room with appropriate air ventilation and lighting. The experimental animals were acclimatized for a week before the experiment. - Administration of the solution to experimental animals weighing > 200 mg was adjusted based on the weight of each rat. Every experimental animal received therapy once a day for three consecutive days at the same time as that on the first day. |
| 1. **Blinding** |  |  |
| Describe who was aware of the group allocation at the different stages of the experiment (during the allocation, the conduct of the experiment, the outcome assessment, and the data analysis). | - | - The authors conducted all stages of the study and were aware of the group allocation during allocation, intervention, outcome assessment, and data analysis. Blinding was not performed due to practical limitations in animal handling. |
| 1. **Outcome measures** |  |  |
| 1. Clearly define all outcome measures assessed (e.g., cell death, molecular markers, or behavioural changes). | 117-118 | - The primary outcome measures were the expressions of TNF-α, NF-κB, IL-8, and MMP-8 in jejunal tissue, which served as indicators of inflammatory response. These molecular markers were assessed using flow cytometry. |
| 1. For hypothesis-testing studies, specify the primary outcome measure, i.e., the outcome measure that was used to determine the sample size. | 119-121 | - The flow cytometry results were analyzed using IBM SPSS Statistic 21 with multivariate analysis of variance (MANOVA) to evaluate the effect of glutamine-arginine combination administration on the number of cells expressing TNF-α, NF-κB, IL-8, and MMP-8. - No single primary outcome measure was predefined. All measured variables, including TNF-α, NF-κB, IL-8, and MMP-8 expression levels, were evaluated with equal importance to assess the overall inflammatory response. |
| 1. **Statistical methods** |  |  |
| 1. Provide details of the statistical methods used for each analysis, including software used. | 119-121 | - The flow cytometry results were analyzed using IBM SPSS Statistics 21 with multivariate analysis of variance (MANOVA) to evaluate the effect of glutamine-arginine combination administration on the number of cells expressing TNF-α, NF-κB, IL-8, and MMP-8. - The analyses were performed assuming the data met the general requirements of MANOVA. |
| 1. Describe any methods used to assess whether the data met the assumptions of thestatistical approach, and what was done if the assumptions were not met. |  |  |
| 1. **Experimental animals** |  |  |
| 1. Provide species-appropriate details of the animals used, including species, strain and substrain, sex, age or developmental stage, and, if relevant, weight. | 73-74 | - This study used healthy and active male Rattus norvegicus Wistar strain rats aged 12–14 weeks, with body weights ranging from 150 to 200 g |
| 1. Provide further relevant information on the provenance of animals, health/immune status, genetic modification status, genotype, and any previous procedures. | 73-76 | - This study used healthy and active male Rattus norvegicus Wistar strain rats aged 12–14 weeks, with body weights ranging from 150 to 200 g, which had never been used in previous experiments. - The rats were obtained from a certified institutional animal facility and were confirmed to be healthy and free from infectious diseases. |
| 1. **Experimental procedures** |  |  |
| 1. What was done, how it was done, and what was used. | 100-112;114-118 | - Sepsis modeling was performed in the positive control group (Group A) and the intervention group (Group B) using LPS 2 ml/kg BW injection, while the negative control group (Group C) received 2 ml/kg BW NaCl 0.9% injection. Injections were performed intraperitoneally on the lower side of the abdomen. - Each group (i.e., Groups A, B, and C) was divided into three groups based on the starting time of therapy, namely 6, 24, and 48 h after LPS or NaCl injections. Dextrose therapy administered to groups A and C consisted of 1 ml of dextrose 5%. Group B received GAC therapy consisting 1:1 of L-glutamine and L-arginine. The dosages adopted from Bakir et al. consisted of 250 mg/kg/d of L-glutamine and 250 mg/kg/d of L-arginine [12]. - GAC was prepared by dissolving 50 mg glutamine and 50 mg arginine in 1 ml of distilled water for rats weighing 200 g, which were administered in a 1 ml syringe. Administration of the solution to experimental animals weighing > 200 mg was adjusted based on the weight of each rat. - Every experimental animal received therapy once a day for three consecutive days at the same time as that on the first day. - The termination process was performed 2 h after the final therapy on the third day. Termination of the experimental animals was carried out by placing the animals inside a sealed container moistened with 5 ml of ether and leaving them for 2-3 min. Rats that were not moving were confirmed dead and dissected to collect the jejunal tissue. - The collected jejunal tissue was examined by flow cytometry to observe the expressions of TNF-α, NF-κB, IL-8, and MMP-8. |
| 1. When and how often | 97-98; 103-84; 111-112 | - The experimental animals were acclimatized for a week before the experiment. - Each group (i.e., Groups A, B, and C) was divided into three groups based on the starting time of therapy, namely 6, 24, and 48 h after LPS or NaCl injections. - Every experimental animal received therapy once a day for three consecutive days at the same time as that on the first day. |
| 1. Where (including detail of any acclimatisation periods) | 94-99 | - Experimental animals were kept at a room temperature inside plastic cages capped with wire mesh and lined with rice husks as bedding. Each cage contained eight rats, equipped with sawdust as feed and clean water. - The cages were placed inside an isolated, locked room with appropriate air ventilation and lighting. The experimental animals were acclimatized for a week before the experiment. Experimental animal conditions and body temperature were measured during the experiment using the murine sepsis score (MSS). |
| 1. Why (provide rationale for procedures) | - | - The LPS-induced sepsis model was selected because it mimics the inflammatory and immune responses observed in human sepsis. Glutamine and arginine combination (GAC) was used for its known anti-inflammatory and immune-supportive effects. The selected time points (6 h, 24 h, and 48 h) were chosen to evaluate the temporal effects of GAC on inflammatory markers. |
| 1. **Results** |  |  |
| 1. Summary/descriptive statistics for each experimental group, with a measure of variability where applicable (e.g., mean and SD, or median and range). | 153-156 | - This study has different findings, where the administration of GAC 24 h after LPS injection resulted in a significant increase in the number of cells expressing IL-8, with a tendency that the group receiving GAC had a higher mean number of cells expressing IL-8 than the control groups. - Summary descriptive statistics (mean ± SD) for each experimental group are presented in Table 1-3 |
| 1. If applicable, the effect size with a confidence interval. | - | - |
